# Soft tissue dimensional change using customized titanium healing abutment in immediate implant placement in posterior teeth

**DOI:** 10.1186/s12903-023-03060-5

**Published:** 2023-06-12

**Authors:** Tanporn Lertwongpaisan, Parinya Amornsettachai, Woraphong Panyayong, Suphachai Suphangul

**Affiliations:** 1 Residency Training Program, Department of Advanced General Dentistry, Faculty of Dentistry, Mahidol University, Bangkok, Thailand; 2 Department of Advanced General Dentistry, Faculty of Dentistry, Mahidol University, Bangkok, Thailand

**Keywords:** Immediate implant placement, Wound healing, Customized titanium healing abutment, Peri-implant mucosa, Soft tissue dimension, Soft tissue management

## Abstract

**Background:**

The morphologic and dimensional alveolar bone is significant for resorption in the first 3 months after tooth removal because they restrict treatment outcomes with respect to function and esthetic. Following teeth extraction, the width and height of the alveolar ridge contour are reduced in both the horizontal and vertical dimensions. Following implant placement, the gingival morphology should be changed minimally compared to pre-extraction. Surrounding natural-like tissue is also an ultimate goal of the dental implant treatment, which is correlated with the cervical third contour on the anatomical tooth, for comfortable cleansing, food impaction avoidance, and esthetics.

**Purpose:**

To evaluate the peri-implant soft tissue changes after immediate implant placement (IIP) with the use of a customized titanium healing abutment in the posterior teeth.

**Method:**

Digital impressions using the intraoral scanner (MEDIT *i*500) were taken from 30 patients. Customized titanium healing abutments were designed and milled before extraction. Flapless extractions were done using surgical guides, 32 immediate implants placement were done in posterior areas, and healing abutments were placed. Soft tissues were scanned during pre-operation, and post-surgery during the 1st, 3rd, and 6th months. A 3D analysis program (Final Surface) evaluated the gingival margin distance, height, contour width, and volume in each period. SPSS was used to analyze the data with a p-value = 0.05. The between-time interval comparisons were done and the analysis was done using a Multivariate test.

**Results:**

Customized titanium healing abutments used in immediate implantation maintained optimal peri-implant mucosa. In intermittent periods, there was no significant reduction in all aspects of the margin distances and heights. During the entire period, the margin height reduction on the buccal, lingual, mesial, and distal was 0.63 mm, 0.93 mm, 0.08 mm, and 0.24 mm, respectively, and contour width reduction on the buccal, lingual, and buccolingual was 0.59 mm, 0.43 mm, and 1.03 mm, respectively. There was a significant reduction in the total buccolingual contour width in the 1st month and total volume in the 3rd to 6th months.

**Conclusions:**

Immediate implant placement with customized titanium healing abutment can achieve the optimal peri-implant mucosa and this protocol is an alternative for soft tissue management.

## Introduction

The morphologic and dimensional alveolar bone is significant for resorption in the first 3 months after tooth removal[1–2] because they restrict treatment outcomes with respect to function and esthetic. Following teeth extraction, the width and height of the alveolar ridge contour are reduced in both the horizontal and vertical dimensions. In particular, the buccal bone wall reduction is more compared to the lingual/palatal wall in the post-extraction socket [1, 3]. Therefore, changes in bone also cause changes in the soft tissues.

Because hard and soft tissue resorptions always occur following tooth extraction, several studies on dental implants have recommended preserving this preoperative structure. In immediate implant placement (IIP) protocol, implant placement is done at the same time as the natural tooth is extracted, which could preserve the buccolingual bone width wider than the delayed implant placement and it is statistically significant at the second surgery [4]. Furthermore, other advantages of IIP include shorter treatment time, fewer surgeries, and a similar survival rate to delayed placement (2-year survival rate of 98.4%) [5] and a flapless approach providing better soft tissue healing [6]. Moreover, implant placement with guided bone regeneration following tooth extraction also induces vertical and horizontal bone gain to compensate for the ridge alterations without complications [7].

Following implant placement, the gingival morphology should be changed minimally compared to pre-extraction. Surrounding natural-like tissue is also an ultimate goal of the dental implant treatment, which is correlated with the cervical third contour on the anatomical tooth, for comfortable cleansing, food impaction avoidance, and esthetics. Thus, using the customized healing abutment could maintain natural gingiva throughout the healing process for oral health, function, and esthetics [8]. “Pseudo cementoenamel junction (Pseudo-CEJ)” design should guide circumferential gingiva to the expected position [9] for the desired prosthesis outcome. Several studies conclude that customized healing abutment offers a better gingival emergence profile than prefabricated healing abutment [10–11].

Dental composite resin, a part of the conventional customized healing abutment, has cytotoxicity caused by substances releasing effects on basic cellular functions, such as cell proliferation and cell viability, that interrupt the wound healing process [12–13]. Considering this, titanium surfaces provide great fibroblastic cell proliferation, are sealed against bacterial leakage, and enhance soft tissue integration and stability [2, 14–15].

IIP with a customized healing abutment is usually used in the anterior areas due to esthetic reasons. This protocol can be applied to the posterior zone using a digital surgical template with customized titanium healing abutment for interdental papilla, gingiva margin, and crestal bone preserving. However, the dimensional change of the soft tissue following this protocol has not yet been evaluated. Hence, this study aimed to evaluate the peri-implant soft tissue changes after IIP with the use of a customized titanium healing abutment in the posterior teeth.

## Method

### Study design and patient selection

A prospective clinical study was performed at the Faculty of Dentistry, Mahidol University in 2019 and 2020. Ethical approval was obtained from the Faculty of Dentistry/Faculty of Pharmacy Mahidol University Institutional Review Board (MU-DT/PY-IRB 2019/035.0706). The overall information was provided to the patients and informed consent was obtained from all eligible patients in this study. One implantologist did all the implant surgical procedures and one prosthodontist did all the prosthetic procedures.

Patients were included with the following criteria: (a) ≥ 18 years of age; (b) medical health status on American Society of Anesthesiologists classification I and II; (c) had at least 1 maxillary or mandibular molar tooth with intact cervical tooth structure and surrounding free gingiva, indicated for extraction for unrestorable tooth reasons (i.e. endodontic failure, unworthy for treatment in deep caries/non-caries lesion and root fracture), and (d) had an adequate vertical bone for immediate implant placement. Patients were excluded with the following criteria: (a) severe acute or chronic periodontitis; (b) soft tissue grafting was needed; (c) smoking ≥ 10 cigarettes/day; (d) history of oral/IV bisphosphonates taking; (e) history of radiotherapy/chemotherapy; (f) clinical or radiographic signs of periapical pathology contraindicating immediate implant placement.

### Customized titanium healing abutment and surgical guide manufacture

**Fig. 1:**
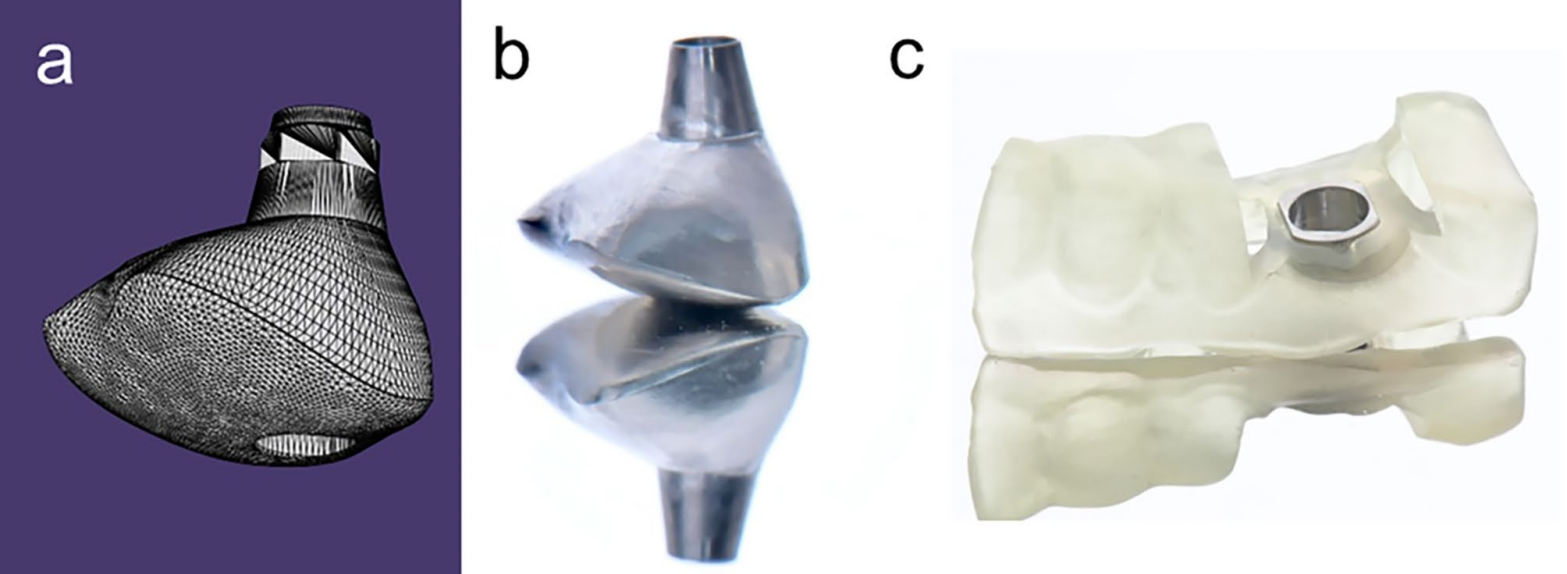
(**a**) The design of customization of healing abutment in form STL (Standard Triangle Language) file format, (**b**) The customized titanium healing abutment was milled from titanium blank, (**c**) The surgical template with a metal sleeve was designed following implantation plan

For each included patient, following medical history, an intraoral examination was done and a digital impression was taken by the intraoral scanner (MEDIT *i*500) for a pre-extraction digital model, and CBCT was taken for prosthetic-driven planning associated with anatomical obstacles, available vertical bone, inferior alveolar nerve, and maxillary sinus. Once the treatment plan was formulated, the dentist sent both STL (Standard Triangle Language) and DICOM (CBCT raw data) files format to the technicians at the Dentium Laboratory produced the digital surgical guide with metal sleeve and surgical protocol using the 3Shape dental system following approval by the dentist. Then, a customized titanium healing abutment was made mimicking the anatomical cervical contour and pseudo-CEJ was made by merging the STL and DICOM digitally and the exterior surface of the healing abutment was designed to resemble a tooth (Fig. 1a). Then, the technicians milled the customized healing abutment digital design from a pre-milled 14 × 14 mm [2] titanium blank with a non-hex connection type, polished it, and sterilized it (Fig. 1b). The digital surgical guide with a metal sleeve back to the dentist (Fig. 1c). The patients were scheduled for implant surgery.

### Operative procedures

**Fig. 2:**
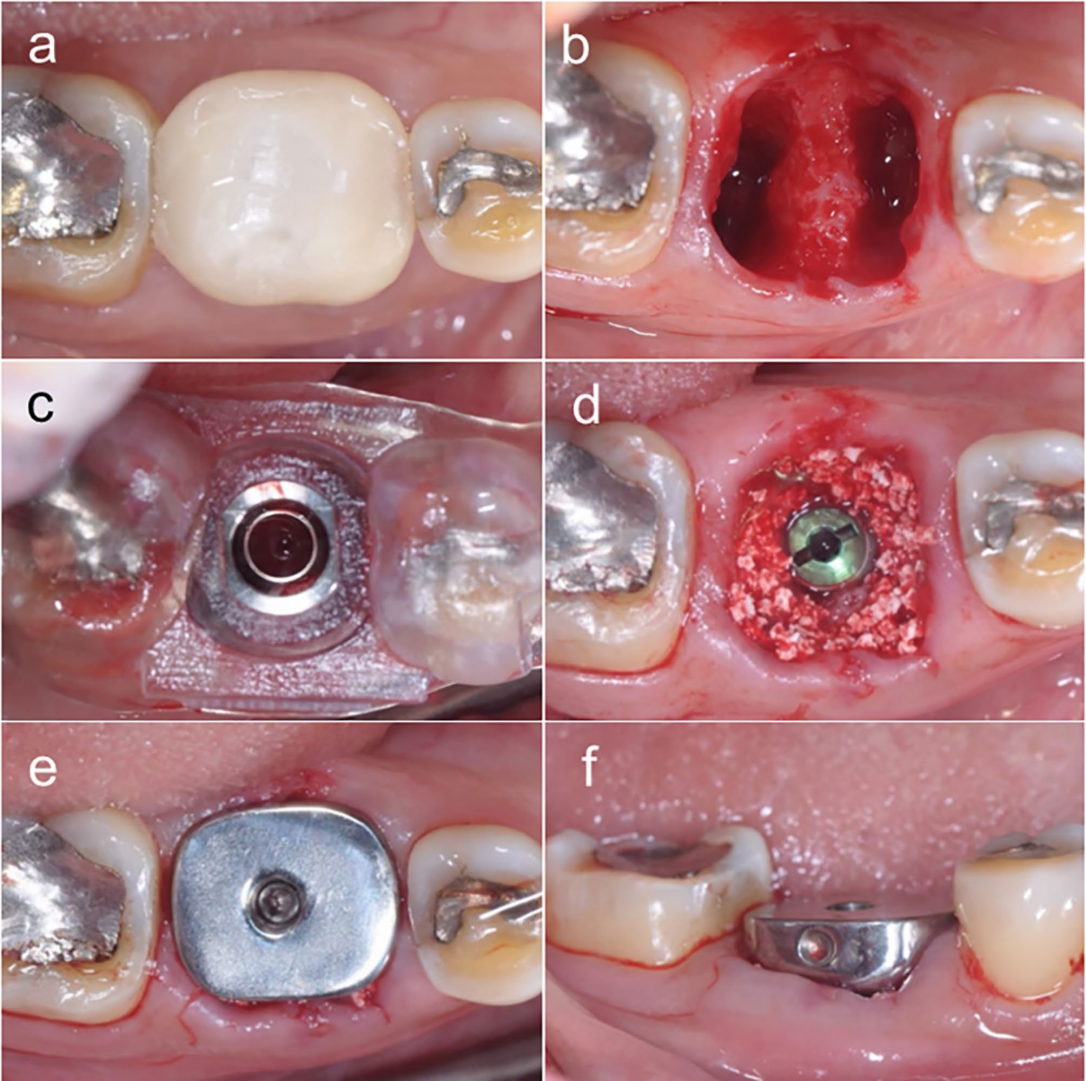
(**a**) Tooth #30 with inadequate restorative tooth structure, (**b**) Tooth #30 was extracted with minimal traumatic technique, (**c**) Complete implantation through a surgical template, (**d**) Alloplastic bone was grafted into the peri-implant gap, (**e**) occlusal view, (**f**) buccal view: The customized titanium healing abutment was connected to the implant fixture and seal on socket wound

Following the injection of local anesthesia in the patient and extraction of the molar was done with minimal traumatic technique without periodontal flap incision and alveolar socket curettage (Fig. 2a and b). Then, the digital surgical guide was placed, and checked the affinity of the reference window with other teeth accurately in the optimal position. The osteotomies were prepared following individual surgical protocol with a surgical-guided surgery kit through a digital surgical guide and drill direction was ensured by radiographic taking. Implant fixture insertion was performed through this digital surgical guide (Fig. 2c). All patients receive a bone-level implant with a coronal connection (SuperLineII, Dentium Co., Suwon, South Korea) but the diameter and length varied in some cases. After implant placement, the operator scanned the surrounding gingiva with a scan body immediately for a post-extraction digital model. Then, the cover screw was placed on the implant fixture, and the spaces between the implant fixture and alveolar bone wall were filled with locally harvested autogenous bone and alloplastic graft (OSTEON™ III, synthetic bone graft material, Genoss Co., South Korea) (Fig. 2d). Finally, the operator removed the cover screw and inserted the customized titanium healing abutment (Fig. 2e and f). Clinically and radiographically, the healing abutment was closely placed on the platform and was occlusion free.

### Post-operative procedures

Post-operative instructions were given and medications prescribed were; Amoxicillin (500 mg, three times daily for 7 days) or clindamycin (300 mg, three times daily for 7 days) for penicillin allergy, Ibuprofen (400 mg, three times daily after meal immediately for 3 days), Acetaminophen (500 mg as needed every 8 h), and 0.12% Chlorhexidine gluconate (500 ml, Gargle twice a day for 7 days).

**Fig. 3:**
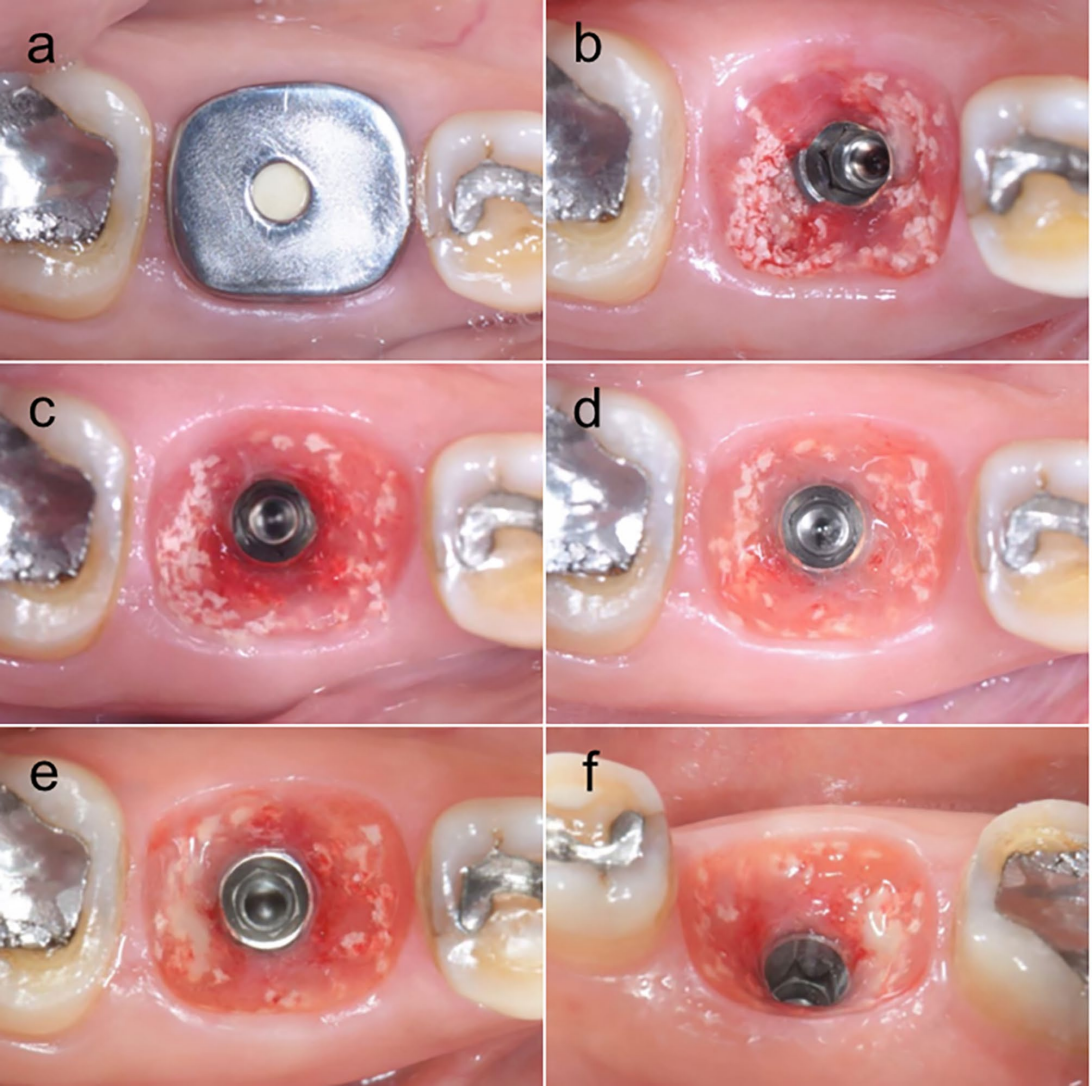
(**a**) The appearance of customized titanium healing abutment and peri-implant mucosa in each following time, (**b**) The peri-implant mucosa in 1st week follow-up, (**c**) The peri-implant mucosa in 1st month follow-up, (**d**) The peri-implant mucosa in 3rd months follow-up, (**e**) occlusal view, (**f**) lingual view: The peri-implant mucosa in 6th months follow-up

**Fig. 4:**
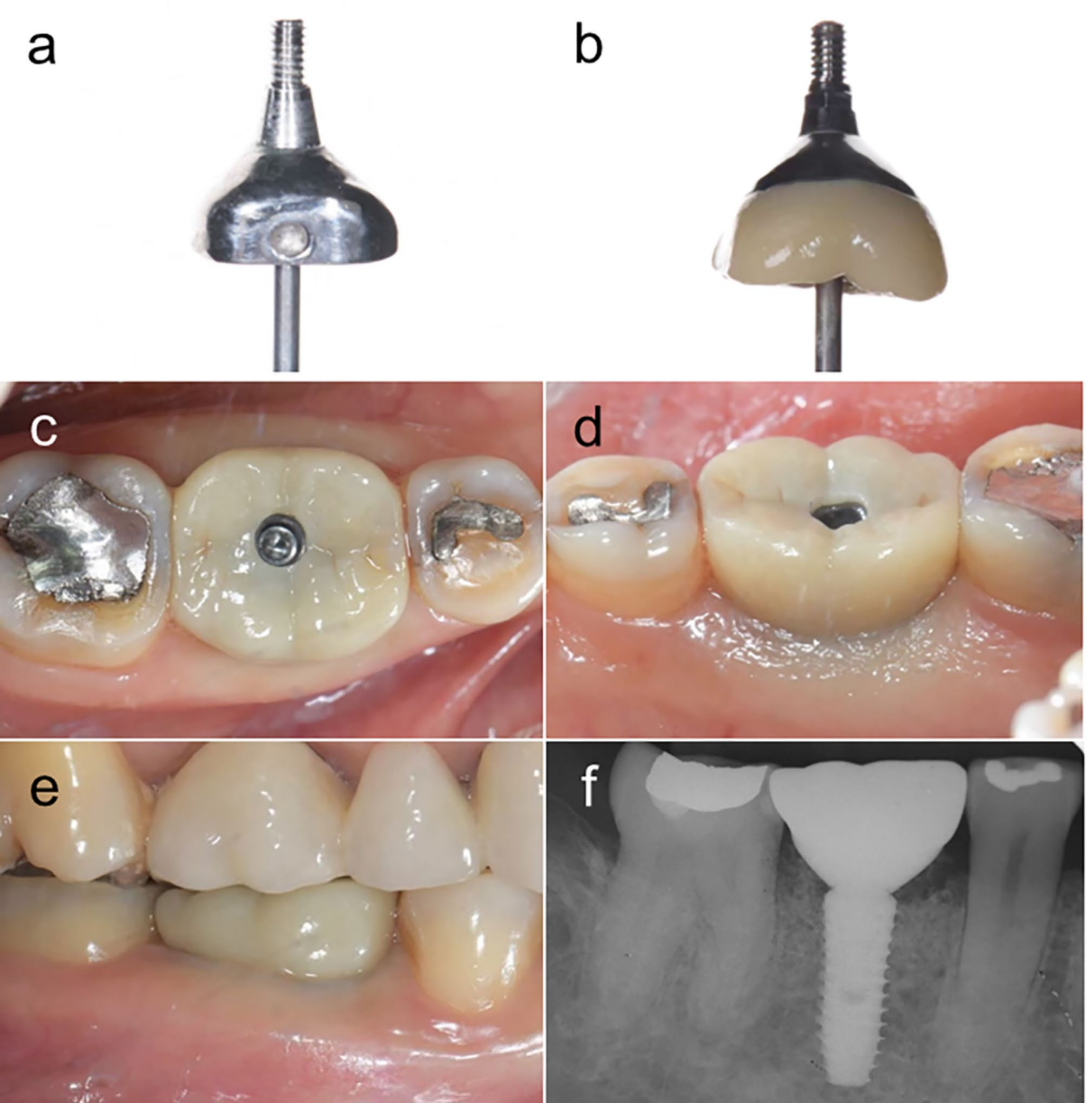
(**a**), (**b**) The outline form comparison between the customized titanium healing abutment and final prosthetic abutment with zirconia crown, (**c**) occlusal view, (**d**) lingual view, (**e**) buccal view, (**f**) radiographic examination: The final screw-retained implant prosthesis was complete after 6 months

All patients were scheduled for follow-up 1 week, 1 month, 3 months, and 6 months after the implant placement. At 1 and 3-month follow-ups, the customized titanium healing abutment was removed (Fig. 3), and irrigated the wound with 0.12% Chlorhexidine gluconate before putting it back and a periapical radiograph was taken. At 6 months, the final impression was made with polyvinyl-siloxane (Variotime, Heraeus Kulzer GmbH, Hanau, Germany), and the customized titanium healing abutment was substituted with the definitive implant restoration (Fig. 4) and recall was done every 6 months.

### Data collection

The implant location and soft tissue in each patient were recorded during the soft and hard tissue wound healing simultaneously by a scanned digital model at a preoperative time, postoperative time, and 1, 3, and 6 months, respectively.

The operator used the intra-oral scanner (MEDIT *i*500) (Medit Corp., Seoul, South Korea) to record implant and peri-implant tissue immediately with a scan body located on the implant platform for digital models each time before putting this healing back.

### Outcome measures

The pre-operation (T_P_), immediate post-operation (T_0_), 1st month (T_1_), 3rd month (T_3_), and 6th month (T_6_) digital models were collected in SLT file with rendering from the MEDIT LINK scanner program and the Final Surface 3D analyzing program was used for model preparation and measurement. Each digital model was superimposed with at least 3 reference points used in matching order. Thus, implant platform planes were placed the same as the X-Y plane, and longitudinal implant lines were put on the same Z-axis.

**Fig. 5:**
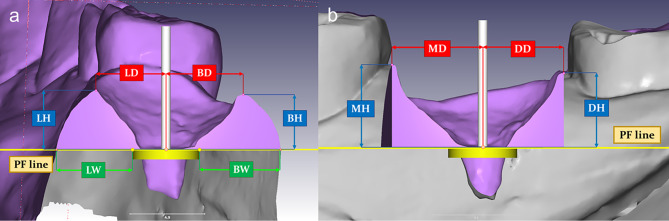
(**a**) Measurement in the buccal-lingual section, (**b**) Measurement in the mesial-distal section

Each matched digital model group was measured for the gingival margin distance, gingival margin height, gingival contour width, and gingival volume in buccolingual (Fig. 5a) and mesiodistal aspects (Fig. 5b). The gingival margin distances were measured from the highest gingival margin point in the buccal, palatal/lingual, mesial, and distal sides to the longitudinal implant line parallelly with the platform plane (buccal margin distance; BD, palatal/lingual margin distance; LD, mesial margin distance; MD and distal margin distance; DD). Gingival heights were measured from the highest gingival margin point in the buccal, lingual, mesial, and distal sides perpendicular to the platform plane (buccal height; BH, palatal/lingual height; LH, mesial height; MH and distal height; DH). The contour widths were measured from the outer surface on the buccal and lingual sides to the implant center on the platform plane (buccal width; BW and palatal/lingual width; LW). Gingival volumes were measured on the gingiva above the platform plane for total gingival volume and separated with a mid mesiodistal plane through the implant center for buccal and lingual gingival volume (total buccolingual volume; BLV, buccal volume; BV and lingual volume; LV).

### Statistical analysis

Statistical analysis was performed to determine an association between the outcome measurement. A statistical software SPSS (Statistics for Windows, version 18.0. IBM Corp., Chicago, Illinois, USA) was used to analyze the data. A p-value < 0.05 was considered statistically significant. The data were recorded at pre-operation, immediate post-operation, and at 1, 3, and 6 months of implant placement and presented as mean and standard error. The between-time interval comparison for parametric continues and data were calculated using the Multivariate test. The null hypotheses were that there was no difference in the peri-implant soft tissue dimension and the volume between pre-operation, immediate post-operation, and at 1, 3, and 6 months of implant placement with the use of a customized titanium healing abutment in molar regions.

## Results

Thirty patients (9 males and 21 females) with a mean age of 49.28 years (range: 23 to 71 years) participated in the study and 32 dental implants were placed. No implants were lost during the 6 months which revealed an overall cumulative implant success rate of 100%. The total number of teeth in this study consisted of 13 maxillary first molars, 11 mandibular first molars, and 8 mandibular second molars. Tooth failure was attributed to fracture (10), furcation perforation (2), endodontic failure (6), and inadequate restorative tooth structure (14). A thick was found in 7 molars and a thin gingival biotype in 25 teeth.

### Model measurement

The intra-class correlation coefficient (ICC) showed good agreement between the repeat measurements in the buccal gingival distance in the 3rd month (T_3_) (range: 0.96–0.99). A cumulative change in the dimension of the soft tissue is shown in Tables 1, 2 and 3.

**Table 1:** Mean ± SE (mm) registered for the changes of gingival margin distance; n = 32 (Max = 13 and Mand = 19)

				Pre-post operation (T_P_-T_0_)	Baseline − 1 month (T_P_-T_1_)	Baseline − 3 months (T_P_-T_3_)	Baseline – 6 months (T_P_-T_6_)	*p*-value^a^
**Buccal distance** **(BD)**	**Total change**	**Maxillary**		0.09 ± 0.14	0.04 ± 0.13	-0.22 ± 0.16	-0.38 ± 0.20	0.063
		**Mandibular**		-0.04 ± 0.24	-0.35 ± 0.23	-0.47 ± 0.26	-0.69 ± 0.24	0.004*
		**All**		0.01 ± 0.15	-0.19 ± 0.15	-0.37 ± 0.17	-0.56 ± 0.16*	< 0.001*
	**Intra-period change**			0.01 ± 0.15	-0.20 ± 0.09	-0.18 ± 0.05*	-0.19 ± 0.07	< 0.001*
**Palatal/** **Lingual** **distance** **(LD)**	**Total change**	**Maxillary**		0.44 ± 0.11*	0.02 ± 0.16	-0.06 ± 0.17	-0.13 ± 0.19	0.022*
		**Mandibular**		-0.44 ± 0.22	-0.68 ± 0.21	-0.71 ± 0.20*	-0.74 ± 0.22*	0.055
		**All**		-0.08 ± 0.16	-0.40 ± 0.15	-0.45 ± 0.15*	-0.49 ± 0.16*	0.012*
	**Intra-period change**			-0.08 ± 0.16	-0.32 ± 0.09*	-0.05 ± 0.05	-0.05 ± 0.04	0.012*
				**Pre-post operation (T**_**P**_ **-T**_**0**_ **)**	**Baseline − 1 month** **(T**_**0**_ **-T**_**1**_ **)**	**Baseline − 3 months** **(T**_**0**_ **-T**_**3**_ **)**	**Baseline – 6** **months** **(T**_**0**_ **-T**_**6**_ **)**	***p*** **-value** ^a^
**Mesial** **distance** **(MD)**	**Total change**	**Maxillary**		NA	0.08 ± 0.07	0.18 ± 0.06	0.09 ± 0.09	0.097
		**Mandibular**		NA	-0.01 ± 0.15	-0.02 ± 0.15	-0.13 ± 0.18	0.334
		**All**		NA	0.03 ± 0.09	0.06 ± 0.09	-0.04 ± 0.11	0.383
	**Intra-period change**			NA	0.03 ± 0.09	0.03 ± 0.05	-0.10 ± 0.06	0.383
**Distal distance** **(DD)**	**Total change**		**Maxillary**	NA	-0.08 ± 0.14	-0.12 ± 0.11	-0.22 ± 0.16	0.524
			**Mandibular**	NA	-0.14 ± 0.11	-0.22 ± 0.12	-0.26 ± 0.13	0.255
			**All**	NA	-0.12 ± 0.09	-0.18 ± 0.08	-0.24 ± 0.10	0.092
	**Intra-period change**			NA	-0.12 ± 0.09	-0.06 ± 0.05	-0.07 ± 0.05	0.092

^a^ Multivariate test. *Statistically significant values (*p*-value < 0.05)

Positive and negative valves indicate gingival margin expansion and contraction, respectively

NA: nonapplicable. The proximal sides could not be identified for pre-operation stage (T_P_).

**Table 2:** Mean ± SE (mm) registered for the changes of gingival margin height; n = 32 (Max = 13 and Mand = 19)

				Pre-post operation (T_P_-T_0_)	Baseline − 1 month (T_P_-T_1_)	Baseline − 3 months (T_P_-T_3_)	Baseline – 6 months (T_P_-T_6_)	*p*-value^a^
**Buccal** **height** **(BH)**	**Total change**	**Maxillary**		-0.57 ± 0.15*	-0.90 ± 0.27*	-0.79 ± 0.33*	-0.83 ± 0.31*	0.010*
		**Mandibular**		-0.42 ± 0.22	-0.73 ± 0.34	-0.79 ± 0.31	-0.86 ± 0.33	0.227
		**All**		-0.48 ± 0.14*	-0.79 ± 0.23*	-0.79 ± 0.22*	-0.85 ± 0.23*	0.017*
	**Intra-period change**			-0.48 ± 0.14*	-0.31 ± 0.16	0.01 ± 0.07	-0.06 ± 0.06	0.017*
**Palatal/** **Lingual** **height** **(LH)**	**Total change**	**Maxillary**		-0.51 ± 0.12*	-0.93 ± 0.16*	-1.01 ± 0.16*	-1.09 ± 0.17*	0.005*
		**Mandibular**		-0.45 ± 0.17	-1.01 ± 0.17*	-1.08 ± 0.17*	-1.22 ± 0.16*	< 0.001*
		**All**		-0.47 ± 0.11*	-0.98 ± 0.12*	-1.05 ± 0.12*	-1.17 ± 0.11*	< 0.001*
	**Intra-period change**			-0.47 ± 0.11*	-0.50 ± 0.11*	-0.07 ± 0.05	-0.12 ± 0.03*	< 0.001*
				**Pre-post operation (T**_**P**_ **-T**_**0**_ **)**	**Baseline − 1 month** **(T**_**0**_ **-T**_**1**_ **)**	**Baseline − 3 months** **(T**_**0**_ **-T**_**3**_ **)**	**Baseline – 6** **months** **(T**_**0**_ **-T**_**6**_ **)**	***p*** **-value** ^a^
**Mesial** **height** **(MH)**	**Total change**	**Maxillary**		NA	0.14 ± 0.20	0.02 ± 0.20	-0.11 ± 0.21	0.338
		**Mandibular**		NA	-0.23 ± 0.22	-0.38 ± 0.24	-0.45 ± 0.23	0.072
		**All**		NA	-0.08 ± 0.16	-0.22 ± 0.17	-0.31 ± 0.16	0.017*
	**Intra-period change**			NA	-0.08 ± 0.16	-0.14 ± 0.06	-0.10 ± 0.05	0.017*
**Distal height** **(DH)**	**Total change**		**Maxillary**	NA	-0.52 ± 0.21	-0.48 ± 0.17	-0.41 ± 0.14	0.122
			**Mandibular**	NA	-0.14 ± 0.20	-0.03 ± 0.19	-0.05 ± 0.19	0.447
			**All**	NA	-0.30 ± 0.15	-0.21 ± 0.14	-0.20 ± 0.13	0.216
	**Intra-period change**			NA	-0.30 ± 0.15	0.09 ± 0.06	0.01 ± 0.07	0.216

^a^ Multivariate test. *Statistically significant values (*p-*value < 0.05)

Positive and negative valves indicate gingival margins higher and lower, respectively

NA: nonapplicable. The proximal sides could not be identified for the pre-operation stage (T_P_).

**Table 3:** Mean ± SE (mm) registered for the changes of gingival contour width; n = 32 (Max = 13, Mand = 19)

			Pre-post operation (T_P_-T_0_)	Baseline − 1 month (T_P_-T_1_)	Baseline − 3 months (T_P_-T_3_)	Baseline – 6 months (T_P_-T_6_)	*p*-value^a^
**Buccal** **width** **(BW)**	**Total change**	**Maxillary**	0.11 ± 0.11	-0.29 ± 0.19	-0.44 ± 0.26	-0.62 ± 0.26	0.045*
		**Mandibular**	0.37 ± 0.12*	-0.27 ± 0.14	-0.49 ± 0.17	-0.75 ± 0.20*	0.001*
		**All**	0.27 ± 0.08*	-0.28 ± 0.11	-0.47 ± 0.14*	-0.70 ± 0.16*	< 0.001*
	**Intra-period change**		0.27 ± 0.08*	-0.55 ± 0.12*	-0.19 ± 0.09	-0.23 ± 0.09	< 0.001*
**Palatal/** **Lingual** **width** **(LW)**	**Total change**	**Maxillary**	0.05 ± 0.07	-0.44 ± 0.17	-0.61 ± 0.19	-0.73 ± 0.20*	0.033*
		**Mandibular**	0.29 ± 0.07*	-0.13 ± 0.09	-0.26 ± 0.13	-0.39 ± 0.13	0.001*
		**All**	0.19 ± 0.05*	-0.26 ± 0.09	-0.40 ± 0.11*	-0.53 ± 0.11*	< 0.001*
	**Intra-period change**		0.19 ± 0.05*	-0.45 ± 0.08*	-0.15 ± 0.05	-0.12 ± 0.03*	< 0.001*
**Sum of width** **(BLW)**	**Total change**	**Maxillary**	0.16 ± 0.16	-0.73 ± 0.33	-1.06 ± 0.43	-1.34 ± 0.43	0.012*
		**Mandibular**	0.66 ± 0.16*	-0.41 ± 0.18	-0.75 ± 0.26	-1.14 ± 0.25*	< 0.001*
		**All**	0.46 ± 0.12*	-0.54 ± 0.17*	-0.87 ± 0.23*	-1.22 ± 0.22*	< 0.001*
	**Intra-period change**		0.46 ± 0.12*	-0.99 ± 0.16*	-0.34 ± 0.12	-0.35 ± 0.10*	< 0.001*

^a^ Multivariate test. *Statistically significant values (*p* < 0.05)

Positive and negative valves indicate gingival margins higher and lower, respectively

### Gingival margin distance

After immediate post-extraction, the buccal margin distance had almost no change in overall (0.01 ± 0.15 mm), maxillary (0.09 ± 0.14 mm), and mandibular (-0.04 ± 0.24 mm) area. The most margin contraction was presented in the intra-period of post-operation to the first month (-0.20 ± 0.09 mm) and the contraction rate declined gradually during the following months until the end of the observation.

Although lingual margin distance had almost no change overall (-0.08 ± 0.16 mm), both the maxillary and mandibular areas had reversed changes in which the maxillary area presented margin expansion (0.44 ± 0.11 mm) while the mandibular area presented shrinkage (-0.44 ± 0.22 mm). The most margin contraction was presented in the intra-period of post-operation to the first month (-0.32 ± 0.09 mm) and after the first month, there was showed very less change in the following months until the end of the observation.

In addition, we found that the mandibular area had a greater influence on the overall change than the maxillary molar in both the buccal and lingual gingival margin distance. The mesial and distal margin distance had very less change without statistically significant from immediate post-extraction to the end of observation.

After 6 months, the total change of buccal, lingual, mesial, and distal gingival margin distance contraction was 0.56 ± 0.16 mm, 0.49 ± 0.16 mm, 0.04 ± 0.11 mm, and 0.24 ± 0.10 mm, respectively.

### Gingival margin height

The buccal and lingual gingival margin shows the most reduction after immediate post-extraction and the first month. After that, there was showed very less change in the following months until the end of the observation. Both maxillary and mandibular areas were changed in the same way.

Although mesial and distal margin height had very less change from immediate post-extraction to the end of observation in the overall aspect (-0.10 ± 0.05), the mesial side was influenced by the mandibular area as the distal side was influenced by the maxillary area.

After 6 months, the total change of buccal, lingual, mesial, and distal gingival margin height reduction was 0.85 ± 0.23 mm, 1.17 ± 0.11 mm, 0.31 ± 0.16 mm, and 0.20 ± 0.13 mm, respectively.

### Gingival contour width

The gingival width on the platform level had expansion after immediate post-extraction, after that the contour started to shrink until the end of observation with the most contraction in the first month (-0.99 ± 0.16 mm) and the shrinkage rate declined gradually during the following months.

After 6 months, the total change of buccal and lingual gingival contour shrinkage was 0.70 ± 0.16 mm and 0.53 ± 0.11, respectively.

## Discussion

Studying the gingival morphology following the implant placement is important for the esthetic outcome of prosthetic rehabilitation. For the esthetic results, gingival morphology following the implant placement should change minimally compared to pre-extraction.

In this clinical study, the null hypotheses were rejected as the peri-implant soft tissue dimension and the volume showed some variations between pre-operation, immediate post-operation, and at 1, 3, and 6 months of implant placement with the use of a customized titanium healing abutment in molar regions. The gingival margin positions slightly changed on all sides. The highest gingival margins reduced apically by approximately 0.85 mm on the buccal side after 6 months, which was predictable after the first month of healing that disagrees with a study by Schropp et al. [1] which demonstrated in the extraction socket healing that buccal margin height slightly increased coronally within the first 3 months. While the maximum change of height in the entire period is a lingual margin of approximately 1.17 mm reduction which agrees with other studies [1, 3, 16–17] which found that the lingual alveolar bone crest had more vertical resorption when compared to the buccal side. From our surgical protocol, the crucial resorption of the lingual bone is caused by implant location which has a closer distance to the lingual than buccal sides following immediate implantation planning following a previous study [18]. In addition, in our study, the mesial and distal margin height did not have a significant vertical change which agrees with previous studies [1, 16–17] because the bulk bone type and minimal traumatic area from a tooth extraction in these regions, unlike the buccal and lingual side.

According to the outcome, the peri-implant mucosa moves apically relate to the bone resorption level. The risk factors of alveolar bone resorption [19] consist of alveolar bone trauma from extraction, thinner bone width, and improper distance between implant location and bone wall. Furthermore, another factor is the difference in the natural gingival fiber pattern between tooth and implant. The gingival fiber around the natural teeth is perpendicular and strongly attached to the tooth surface while this runs parallel to the smooth surface around the implant and the connective tissue form to be a network of interconnecting fibers that may lead to peri-implant mucosa contraction and collapse in the vertical axis [20].

In a previous study [21], the alveolar bone between the implant platform and the outer buccal and lingual/palatal bone wall was determined following 4 months of healing and found that the reduction of the height of the walls was more pronounced at the buccal than at the lingual aspect of the extraction socket. The horizontal resorption of the buccal and lingual/palatal bone width was 56% and 30%, respectively [16] and the ridge preservation protocol with allograft has less buccolingual alveolar width reduction compared to spontaneous healing [22]. Another study [1] stated that bone and soft-tissue contour changes following tooth extraction and found that ridge width has a 2/3 reduction occur in the first 3 months and has 50% remained alveolar ridge reduction after 12 months of healing. While our outcome found that the buccal and lingual/palatal gingival width reductions were 10.58% and 7.01%, respectively after immediate implant placement with bone grafting in the peri-implant gap at the 6th month. In another study [22], it was stated that the height and width of the residual ridge can be maintained by ridge preservation procedure using FDBA and a collagen membrane when compared to the untreated alveolar socket and the study [23] which stated that bone substitute filling in the peri-implant gap had a lesser gingival buccolingual width reduction than the unfilled gap in the immediate implant placement. Thus, the immediate implant placement with bone substitution is the main reason in our protocol that may create peri-implant mucosa architecture as original as possible when compared with the extraction sides without ridge preservation.

In posterior teeth, the acquisition of the attached gingiva is important because of effective cleaning. The emergence profile plays a major role in implant sustainability that indicates implant esthetics score and sealing ability from bacterial invasion [24] The emergence profile of the implant prosthesis is associated with the gingiva relationship between height and thickness of peri-implant tissues. We measured gingival height and thickness on the digital model in the 6th month and found that the ratio is 1:2.72. While other studies showed that the measurement of the ratio in both the anterior and posterior teeth was 1:1.5 [25] and 1:1.19 [26 on the poured stone cast. Compared to a previous study, our study only collected in the molar region which agrees with another study [25] that reported that the gingival width was larger in the posterior region than in the anterior region, and the diameter of the implant was not related to the biological height-width ratio.

The healing abutment type is also involved in the management of the peri-implant tissue. In general, standard circular healing abutments are usually the first choice but this study [9] stated that this abutment type is unable to create a proper emergence profile. The customization of the healing abutment by incrementally adding flowable composite on the temporary implant abutment may offer an alternative for the guided soft tissue healing that could be a scaffold to promote the shape of the peri-implant mucosa and eliminate second-stage surgery but resin monomer has cytotoxicity by substances releasing effect on basic cellular functions, such as cell proliferation and cell viability, to interrupt the wound healing process [12]. Thus, titanium alloy should be the material of choice for healing abutment which was snuggled up to fresh wounds because it provided great fibroblastic cell proliferation, sealed against bacterial leakage, and enhanced soft tissue integration and stability [2].

Our study introduced intra-oral scanning and digital data measurement as new methods for assessing the dimensional changes of soft tissue during the healing period in one-step recording direct from the source and ensured this measurement protocol is more accurate than the conventional step which cumulates routine error in the impression taking with weight material, the poured stone model and hand-manual measuring. However, in a previous study [27], it was concluded that polyvinyl siloxane full-arch impression had more accuracy than intraoral scanning. Medit link intraoral scanner is a new program therefore there is no evidence of support for in vitro and in vivo studies.

Customization of the healing abutment had more dimensional improvement in soft tissue healing when compared with stock healing abutment [28]. First, because of flapless protocol, a stock healing abutment is unable to completely seal the underlying alveolar socket in the immediate implantation. If this abutment can’t be avoided, using a flap elevation procedure is needed to seal the grafting material. [29] Second, the mismatch between the stock healing abutment and the emergence profile in the molar region is caused by improper prosthetic abutment design or more time spent contouring gingival tissues before the final customized prosthetic abutment. Our study protocol required consecutive disconnections of the customized healing abutments, This protocol didn’t cause peri-implant mucosa damage and negative dimensional changes when compared with normal workflow (2-time disconnection) [30] and didn’t cause bone loss compared with normal workflow (3-time disconnection) [31].

The customized healing abutment must determine its final position before the actual implant was placed following the digital planning. Thus, the placed implant must be precisely located on the horizontal and vertical axis. The surgical guided template was necessary for our protocol because a recent study [32] compared the preciseness between several types of the static guided template and freehand implant surgery, and stated that all guided protocols had less incidence of misangulation of the implant than freehand protocol. For this reason, the precision of implant location is important not only for the customized healing position in our study but also associated with the prosthetic plan and esthetic outcome.

The limitation of our study is that we were unable to match digital models each time in the final surface software by using the scan body because the platform seating of the scan body is made from plastic that was distorted following the amount of driver torque. For this reason, if we try to match each digital model together, we would notice the incomplete superimposing of the scan body in the longitudinal axis only. We suggest for the platform seating of the scan body have a titanium surface and the torque valve should be set to eliminate material distortion and avoid implant platform damage. Although the customized titanium healing abutments in each case were designed from STL and DICOM files superimposing together, we found minimal error contour of customization because our result on the gingival margin height and distance at the mesial side showed gain during 3rd months of healing. For this reason, this error caused complexity of the merged proximal area with the adjacent tooth and therefore it was difficult to mimic the outer tooth surface in the DICOM file.

## Conclusions

Although dimensional reduction of the peri-implant mucosa cannot be inhibited during soft tissue healing, immediate implant placement with customized titanium healing abutment may help to reduce the dimensional change of the peri-implant mucosa. This clinical protocol can be used for soft tissue management in implant dentistry. Within the limitations of this study, this research can be continued for a longer follow-up to evaluate peri-implant mucosa in the long term.

## Data Availability

The dataset used in the current study is available from the corresponding author on reasonable request.
